# Clinical Prediction of Functional Decline in Multiple Sclerosis Using Volumetry-Based Synthetic Brain Networks

**DOI:** 10.3390/life16030459

**Published:** 2026-03-11

**Authors:** Alin Ciubotaru, Alexandra Maștaleru, Thomas Gabriel Schreiner, Cristiana Filip, Roxana Covali, Laura Riscanu, Robert-Valentin Bilcu, Laura-Elena Cucu, Sofia Alexandra Socolov-Mihaita, Diana Lăcătușu, Florina Crivoi, Albert Vamanu, Ioana Martu, Lucia Corina Dima-Cozma, Romica Sebastian Cozma, Oana-Roxana Bitere-Popa

**Affiliations:** 1 Grigore T. Popa University of Medicine and Pharmacy, 700115 Iasi, Romania; alinciubotaru94@yahoo.com (A.C.); alexandra.mastaleru@umfiasi.ro (A.M.); thomas.schreiner@umfiasi.ro (T.G.S.); cristiana.filip@umfiasi.ro (C.F.); ioana.martu@umfiasi.ro (I.M.); cozma.dima@umfiasi.ro (L.C.D.-C.); sebastian.cozma@umfiasi.ro (R.S.C.); oana-roxana.bitere@umfiasi.ro (O.-R.B.-P.); 2 Neurology Clinic, “N. Oblu” Clinical Emergency Hospital, 700309 Iasi, Romania; 3 Department of Radiology, Biomedical Engineering Faculty, Grigore T. Popa University of Medicine and Pharmacy, 700115 Iasi, Romania; 4 Department of Morphofunctional Sciences, Grigore T. Popa University of Medicine and Pharmacy, 700115 Iasi, Romania; laura.knieling@umfiasi.ro; 5 Doctoral School, Grigore T. Popa University of Medicine and Pharmacy, 700115 Iasi, Romania; robert-valen-tin.bilcu@d.umfiasi.ro (R.-V.B.); dudau.laura-elena@d.umfiasi.ro (L.-E.C.); 6 Doctoral School, Carol Davila University of Medicine and Pharmacy, 020021 Bucharest, Romania; sofia.socolov@yahoo.com; 7 Department of Pharmacological Phisics, Grigore T. Popa University of Medicine and Pharmacy, 700115 Iasi, Romania; diana.lacatusu@umfiasi.ro (D.L.); florina.crivoi@umfiasi.ro (F.C.); 8 Basic and Clinical Neuroscience Department, Institute of Psychiatry, Psychology and Neuroscience, King’s College London, London SE5 8AF, UK; albert.vamanu@kcl.ac.uk

**Keywords:** network vulnerability, disability severity, clinical progression, machine learning models, inter-hemispheric connections

## Abstract

Background: Disability progression in multiple sclerosis (MS) is increasingly recognized as a consequence of large-scale brain network disruption rather than isolated regional damage. Although diffusion tensor imaging (DTI) is the reference method for assessing structural connectivity, its limited availability restricts widespread clinical application. There is therefore a critical need for alternative approaches capable of capturing network-level alterations using routinely acquired MRI data. Objective: This study aimed to determine whether synthetic structural connectivity matrices derived from standard regional volumetric MRI can capture clinically meaningful network alterations in MS and predict subsequent functional progression, particularly upper limb decline. Methods: Regional brain volumetry was obtained from routine T1-weighted MRI using an automated, clinically approved volumetric pipeline. Synthetic structural connectivity matrices were generated by integrating principles of structural covariance, distance-dependent connectivity, and disease-specific vulnerability patterns. Graph-theoretical network metrics were extracted to characterize global and regional topology. Machine learning models including logistic regression, support vector machines, random forests, and gradient boosting were trained to predict clinical progression defined by worsening on the 9-Hole Peg Test. Dimensionality reduction was performed using principal component analysis, and model performance was evaluated using balanced accuracy, AUC-ROC, and resampling-based validation. Feature importance analyses were conducted to identify network vulnerability patterns. Results: Synthetic connectivity networks exhibited biologically plausible properties, including preserved but attenuated small-world organization. Global efficiency showed a strong inverse correlation with disability severity (EDSS). Patients with clinical progression demonstrated marked reductions in network integration and segregation, alongside increased characteristic path length. Machine learning models achieved robust prediction of upper limb functional decline, with ensemble-based methods performing best (balanced accuracy > 80%, AUC-ROC up to 0.85). A limited subset of connections accounted for a disproportionate share of predictive power, predominantly involving frontoparietal associative networks, thalamocortical pathways, and inter-hemispheric connections. In a longitudinal subset, network-level alterations preceded measurable clinical deterioration by several months. Conclusions: Synthetic structural connectivity derived from routine volumetric MRI captures clinically relevant network-level disruption in multiple sclerosis and enables accurate prediction of functional progression. By bridging network neuroscience with widely accessible imaging data, this framework provides a pragmatic alternative for connectomic analysis when diffusion imaging is unavailable and supports a network-based understanding of disease evolution in MS.

## 1. Introduction

Multiple sclerosis (MS) is a chronic inflammatory and neurodegenerative disorder of the central nervous system characterized by complex interactions between focal demyelinating lesions, diffuse axonal injury, and progressive neurodegeneration [1]. While conventional magnetic resonance imaging (MRI) measures such as lesion burden and global or regional brain atrophy play a central role in diagnosis and disease monitoring, their ability to explain clinical heterogeneity and predict functional outcomes remains limited [2]. Increasing evidence suggests that disability progression in MS reflects not only localized tissue damage but also large-scale disruption of brain network organization.

Over the past decade, the application of connectomics has substantially advanced our understanding of MS as a network disorder. Structural and functional connectivity studies have demonstrated that clinical manifestations particularly cognitive impairment and upper limb dysfunction are more strongly associated with network-level alterations than with isolated regional pathology [3]. In this framework, MS-related damage is increasingly conceptualized as a distributed process affecting highly connected hub regions and long-range integrative pathways.

Diffusion tensor imaging (DTI) is currently regarded as the reference standard for in vivo assessment of structural connectivity through reconstruction of white matter fiber pathways [4]. However, despite its methodological strengths, DTI has important practical limitations. It requires specialized acquisition protocols, extended processing pipelines, and substantial computational resources, restricting its use in routine clinical practice and limiting its availability in large retrospective datasets [5]. Consequently, a substantial proportion of clinically relevant MRI data remains underutilized for network-level analyses.

Recent advances in network neuroscience have fundamentally transformed our understanding of multiple sclerosis as a disconnection syndrome. Several large-scale studies have demonstrated that structural and functional brain networks are extensively disrupted in MS, with damage preferentially affecting highly connected hub regions such as the thalamus, default-mode network, and frontoparietal networks. Schoonheim and colleagues proposed the concept of network vulnerability, whereby highly connected hubs are particularly susceptible to pathological damage due to their metabolic demands and centrality. This framework suggests that monitoring network integrity may be more clinically relevant than assessing focal lesion burden alone.

Diffusion tensor imaging studies have consistently shown that global network efficiency is reduced in MS patients compared to healthy controls, and that these reductions correlate with both physical disability and cognitive impairment. Furthermore, recent work has demonstrated that structural disconnection in subcortical regions, particularly the thalamus, represents an important determinant of both disability progression and cognitive impairment in MS. However, despite the prognostic value of diffusion-based connectivity measures, their clinical translation remains limited. Advanced diffusion protocols require specialized acquisitions, lengthy scan times, and complex post-processing pipelines that are not feasible in routine clinical practice [5]. This limitation has motivated the search for alternative approaches that can approximate network-level information from routinely acquired MRI sequences.

In MS specifically, recent work has shown that volumetric alterations follow network-constrained patterns, with coordinated atrophy occurring in anatomically connected regions [6]. These findings provide the biological foundation for inferring connectivity from volumetric data.

The present study builds upon this growing body of evidence by introducing a synthetic connectivity framework that integrates principles of structural covariance, distance-dependent connectivity, and disease-specific vulnerability patterns. By doing so, we aim to determine whether network-level information derived from routine volumetric MRI can predict functional progression with accuracy comparable to diffusion-based approaches, thereby bridging the gap between advanced network neuroscience and clinical practice.

In contrast, regional brain volumetry derived from standard T1-weighted MRI is widely available, robust, and routinely acquired in clinical care [6]. Volumetric measures have demonstrated prognostic relevance in MS, with specific patterns of cortical and subcortical atrophy predicting future disability and cognitive decline [7,8]. Nevertheless, volumetry alone provides limited insight into the relational architecture of the brain that is, how regional changes co-occur and interact within distributed networks.

Structural covariance analysis offers a conceptual bridge between volumetric data and network neuroscience. Prior studies have shown that correlations in regional brain volumes across individuals reflect underlying anatomical connectivity, shared developmental trajectories, and common vulnerability to pathological processes [9,10,11]. In neurodegenerative and inflammatory disorders, regions that are structurally connected tend to exhibit coordinated atrophic changes, suggesting that volumetric similarity can serve as an indirect marker of network integrity.

Building on this theoretical foundation, we propose that biologically informed synthetic structural connectivity matrices can be generated from individual-level volumetric data. By incorporating principles of structural covariance, distance-dependent connectivity, and disease-specific vulnerability patterns, such synthetic connectomes may approximate meaningful aspects of structural network organization in MS. Importantly, this approach has the potential to extend network-level analyses to clinical datasets lacking diffusion imaging.

Over the past two decades, advanced MRI techniques have substantially expanded our understanding of MS pathology beyond conventional lesion assessment. Diffusion tensor imaging studies have consistently demonstrated microstructural white matter abnormalities correlating with disability [4,5]. Resting-state fMRI has revealed widespread functional connectivity disruptions in default mode, sensorimotor, and visual networks [1]. Volumetric MRI has established gray matter atrophy particularly thalamic and cortical as a powerful predictor of long-term disability accumulation [6]. Recent work combining multi-shell DTI with clinical assessment can distinguish early MS patients from healthy controls [3], while spinal cord atrophy has emerged as one of the strongest predictors of disability progression independent of relapse activity [4]. Machine learning approaches integrating multiple MRI modalities with serum biomarkers have identified distinct MS subtypes with different progression trajectories [7]. Despite these advances, clinical translation of many advanced techniques remains limited by specialized acquisition requirements [5], motivating the search for approaches that extract network-level information from routinely acquired MRI sequences the central rationale for the synthetic connectivity framework presented in this study.

By enabling early identification of patients at risk for functional decline using routinely acquired imaging data, this study contributes directly to improving therapeutic strategies in multiple sclerosis. First, the ability to predict upper limb progression with high accuracy allows clinicians to consider earlier escalation of disease-modifying therapies for high-risk patients, potentially preventing irreversible disability. Second, the identification of specific vulnerable networks such as frontoparietal and thalamocortical pathways can guide targeted rehabilitation interventions, directing occupational therapy and cognitive training to patients most likely to benefit. Third, the finding that network alterations precede clinical symptoms by several months positions synthetic connectivity as a tool for stratifying patients in clinical trials of neuroprotective agents, enriching study populations with individuals at imminent risk of progression. Finally, by extracting network-level information from standard T1-weighted MRI, this framework democratizes access to advanced prognostic assessment, enabling its implementation in centers without access to specialized diffusion imaging and supporting more equitable, personalized care across diverse clinical settings.

### 1.1. Study Aim

The aim of this study is to investigate whether synthetic structural connectivity derived from routinely available regional volumetric MRI data can capture clinically meaningful network-level alterations in multiple sclerosis and predict subsequent functional progression. Specifically, the study seeks to determine whether network representations inferred from volumetric information can serve as reliable proxies for structural disconnection and provide prognostic insight into disease evolution in the absence of diffusion-based imaging.

### 1.2. Study Objectives

#### 1.2.1. Primary Objective

To evaluate the predictive value of synthetic structural connectivity matrices, generated from regional brain volumetry, for identifying patients with multiple sclerosis at risk of clinically significant upper limb functional decline, as measured by progression on the 9-Hole Peg Test.

#### 1.2.2. Secondary Objectives

1. To characterize the global and regional topological properties of synthetic connectivity networks in patients with multiple sclerosis.
2. To assess the relationship between synthetic network metrics and established clinical measures of disability, including EDSS.
3. To identify specific network connections and brain regions that disproportionately contribute to functional progression.
4. To compare the performance of different machine learning algorithms in modeling progression risk based on synthetic connectivity features.
5. To explore the temporal relationship between network-level disruption and subsequent clinical deterioration in a longitudinal patient subset.

##### Relationship Between Pathophysiology and the Need for Connectivity-Based Investigation

The complex pathophysiology of multiple sclerosis characterized by inflammatory demyelination, axonal transection, and progressive neurodegeneration provides the fundamental rationale for investigating brain connectivity in this disease. This is because these pathological processes disrupt the intricate network of connections that enable communication between brain regions, and this disruption may be more closely linked to clinical disability than focal damage alone.

Multiple sclerosis affects both white and gray matter compartments of the central nervous system. Although focal lesions represent a hallmark of the disease, accumulating evidence indicates that long-term disability and functional impairment are only weakly correlated with lesion burden and regional tissue loss alone. Instead, clinical manifestations reflect the cumulative impact of distributed pathological processes that disrupt communication between anatomically and functionally interconnected brain regions.

From a pathophysiological standpoint, MS-related damage propagates along large-scale neural systems rather than remaining confined to isolated regions. Cortical and subcortical atrophy, thalamic involvement, and corpus callosum degeneration exemplify how structural damage affects integrative hubs that coordinate information transfer across networks. Such network-level disorganization provides a mechanistic explanation for the frequent dissociation between focal imaging findings and clinical symptoms.

Structural connectivity constitutes the anatomical substrate of these distributed interactions. Disruption of white matter pathways impairs the efficiency, redundancy, and resilience of brain networks, leading to reduced functional integration and compensatory reorganization. Consequently, assessment of connectivity is essential for understanding how diffuse pathological changes translate into clinical disability. However, comprehensive evaluation of structural connectivity remains challenging in routine practice due to the limited availability of advanced diffusion imaging.

This pathophysiological context underscores the necessity of developing alternative methods capable of capturing network-level disconnection using clinically accessible imaging modalities. Inferring connectivity from regional volumetric relationships represents a biologically grounded strategy to address this need, particularly in diseases such as MS where neurodegeneration follows network-constrained patterns.

### 1.3. Gaps in the Existing Literature

Despite substantial advances in neuroimaging and network neuroscience, several critical gaps remain in the study of connectivity in multiple sclerosis. First, the majority of studies examining structural connectivity in MS rely on diffusion tensor imaging or multimodal imaging protocols that are not universally available and are often absent from retrospective or real-world clinical datasets. This limits the translational applicability of network-based findings to everyday clinical practice.

Second, volumetric MRI metrics and connectivity analyses are frequently treated as separate domains of investigation. While volumetric measures are well established as markers of neurodegeneration and disease progression, their potential to inform network-level models has not been fully exploited, despite strong theoretical support from structural covariance research.

Third, relatively few studies have focused on the predictive utility of connectivity measures for future clinical progression, particularly using approaches that can be generalized beyond specialized imaging techniques. Existing work often emphasizes cross-sectional associations rather than longitudinal prognostic value.

Finally, current connectivity models rarely integrate disease-specific vulnerability patterns into their analytical frameworks. MS is known to preferentially affect certain networks, such as frontoparietal and thalamocortical systems, yet many network analyses treat all connections equivalently, potentially obscuring clinically relevant heterogeneity.

By addressing these gaps, the present study aims to contribute a methodologically and clinically relevant framework that aligns pathophysiological understanding with accessible neuroimaging data and predictive modeling.

In the present study, we introduce a novel framework for generating synthetic structural connectivity matrices from routine volumetric MRI in patients with MS. We evaluate whether these synthetic connectomes exhibit biologically plausible network properties and whether they can predict clinically relevant disease progression. Using machine learning techniques, we assess the ability of synthetic connectivity features to predict upper limb functional decline as measured by the 9-Hole Peg Test (9HPT). Finally, we examine network vulnerability patterns to identify specific regions and connections associated with functional deterioration.

Specifically, this study addresses three key questions:

(1) Can synthetic connectivity matrices derived from regional volumetry capture meaningful network-level properties in MS?
(2) Do these synthetic connectomes enable accurate prediction of clinical progression?
(3) Which network components are preferentially vulnerable in patients who experience functional decline?

## 2. Materials and Methods

### 2.1. MRI Acquisition and Volumetric Processing

Image processing was performed using the “mdbrain” software (version 2.2) from Mediaire GmbH, Berlin, Germany, which is approved in accordance with the European Medical Devices Directive. The “mdbrain” software has been approved as a medical device in accordance with European Commission requirements. It performs automated brain volumetry for different brain segments or lobes using native T1-weighted 3D MRI sequences. The system uses a custom deep learning segmentation model based on the “U-Net” architecture to perform brain volumetry studies more quickly. The brain volumes of 42 brain regions, including the hippocampus, are measured using percentiles and compared with a cohort of healthy individuals (*n* = 6371, age range 10–97), controlling for age, sex, and total intracranial volume (ICV). The volumes measured include total brain volume (TBV), gray matter (GM), white matter (WM), and cortical gray matter (cGM). At the brainstem level, separate measurements are performed for the midbrain and pons, and at the ventricular level.

#### 2.1.1. Study Population and Clinical Assessment

This retrospective cohort study included patients with a definite diagnosis of multiple sclerosis (MS) recruited from the Neurology Clinic of Clinical Rehabilitation Hospital Iasi, Romania, between January 2020 and December 2024. The diagnosis of MS was established according to the revised 2017 McDonald criteria. The study was approved by the Ethics Committee of the **Clinical Rehabilitation Hospital Iasi** (approval no. 22/16 November 2022) and the Research Ethics Committee of “Grigore T. Popa” University of Medicine and Pharmacy Iasi (approval no. 265/1 February 2023). Written informed consent was obtained from all participants prior to enrollment.

**Inclusion criteria** were: (1) age between 18 and 70 years; (2) a confirmed diagnosis of relapsing-remitting MS (RRMS) or secondary progressive MS (SPMS); (3) availability of a high-quality baseline 3D T1-weighted MRI scan performed as part of routine clinical care; (4) completion of a clinical assessment, including the Expanded Disability Status Scale (EDSS) and 9-Hole Peg Test (9HPT), within three months of the MRI scan; and (5) at least one follow-up clinical assessment after a minimum of 12 months to determine progression status.

**Exclusion criteria** were: (1) presence of other significant neurological disorders (e.g., stroke, neurodegenerative diseases, traumatic brain injury); (2) major psychiatric disorders (e.g., schizophrenia, bipolar disorder) or active severe depression; (3) history of alcohol or substance abuse; (4) contraindications to MRI (e.g., claustrophobia, ferromagnetic implants, pregnancy); (5) relapse or corticosteroid treatment within the four weeks preceding the baseline MRI and clinical assessment; and (6) poor image quality precluding automated volumetric analysis.

**Clinical and demographic data** collected at baseline included: age, sex, disease duration, disease phenotype (RRMS or SPMS), EDSS score, 9HPT time for both hands, and current disease-modifying therapies (DMTs). All medications were recorded, including specific DMTs (interferon-beta, glatiramer acetate, dimethyl fumarate, fingolimod, natalizumab, ocrelizumab, cladribine) and symptomatic treatments for spasticity, fatigue, or neuropathic pain.

#### 2.1.2. Study Design and Progression Assessment

This study employed a **longitudinal retrospective cohort design**. Clinical and imaging data were collected at baseline (time of MRI acquisition), and patients were followed prospectively for a minimum of 12 months to assess functional progression. The follow-up period extended up to 24 months for a subset of patients, with clinical assessments repeated at approximately 12-month intervals as part of routine clinical care.

Progression status was determined based on the change in 9-Hole Peg Test (9HPT) performance between baseline and the last available follow-up assessment. Patients were classified as **progressors** if they met the following criterion:

A confirmed increase of **≥20%** in the time required to complete the 9HPT with either the dominant or non-dominant hand, compared to baseline, sustained at the 12-month follow-up visit.

This threshold was chosen based on previously published criteria defining clinically meaningful worsening in upper limb function in multiple sclerosis (references). Patients who did not meet this criterion, or who showed improvement or stability in 9HPT performance, were classified as **non-progressors**.

For patients with multiple follow-up visits, the 12-month assessment was used as the primary time point for classification to ensure consistency across the cohort. In the longitudinal subset with extended follow-up (up to 24 months), additional analyses were performed to explore the temporal relationship between baseline network metrics and subsequent clinical decline.

### 2.2. Statistical and Machine Learning Analysis

Given the high dimensionality of connectivity-derived features and the complex, non-linear relationship between brain network organization and clinical progression in MS, a multi-layered statistical and machine learning framework was employed. This approach was designed to ensure robustness, control for confounding factors, and maximize interpretability while minimizing overfitting.

### 2.3. Descriptive and Inferential Statistical Analysis

Baseline demographic, clinical, and imaging variables were first examined using conventional statistical methods to characterize the study population and to identify potential confounders. Continuous variables were assessed for normality using visual inspection and distributional measures. Depending on data distribution, between-group comparisons were conducted using either Student’s *t*-test or the Mann–Whitney U test. Categorical variables were compared using the χ^2^ test or Fisher’s exact test, as appropriate.

These initial analyses served two purposes. First, they ensured comparability be-tween progressor and non-progressor groups. Second, they identified variables such as baseline disability or total brain volume that could influence connectivity measures and therefore required adjustment in subsequent analyses.

To account for the influence of demographic and disease-related covariates on network metrics, group comparisons of connectivity-derived measures were performed using analysis of covariance (ANCOVA), adjusting for age, sex, and disease duration. This step was essential to isolate the effects attributable specifically to network alterations rather than to known confounding factors.

Correlation analyses were conducted to explore relationships between connectivity metrics and clinical outcomes. Pearson or Spearman correlation coefficients were used depending on variable distributions. These analyses provided an interpretable link between network properties and clinical progression, supporting the biological relevance of the synthetic connectivity framework.

### 2.4. Rationale for Machine Learning-Based Prediction

Clinical progression in MS is inherently multifactorial and cannot be adequately captured by single imaging or clinical variables. Connectivity-derived features are numerous, interdependent, and often exhibit non-linear interactions. Under these conditions, traditional regression approaches are limited by assumptions of linearity and independence.

Machine learning (ML) techniques were therefore employed to model complex relationships between synthetic connectivity features and clinical progression. ML methods are particularly well suited for high-dimensional neuroimaging data, as they can accommodate multicollinearity, identify higher-order interactions, and learn predictive patterns without prespecified hypotheses.

### 2.5. Feature Dimensionality Reduction

Synthetic connectivity matrices yielded several thousand potential features, including individual connection strengths, regional network metrics, and global graph-theoretical measures. Directly inputting this feature space into predictive models would substantially increase the risk of overfitting and reduce generalizability.

To address this challenge, principal component analysis (PCA) was applied as an unsupervised dimensionality reduction technique. PCA preserves the maximal amount of variance while transforming correlated variables into orthogonal components. Components explaining 95% of the total variance were retained, resulting in a compact feature representation that preserved network-level information while reducing noise and redundancy. Importantly, PCA was performed using training data only to prevent information leakage.

### 2.6. Model Selection and Training Strategy

Four supervised machine learning algorithms were selected to represent complementary learning paradigms:

• Logistic Regression, as a linear baseline model providing interpretability and probabilistic outputs;
• Support Vector Machine (SVM) with a linear kernel, suitable for high-dimensional data and robust to overfitting;
• Random Forest, an ensemble-based, non-linear model capable of capturing complex feature interactions;
• Gradient Boosting, which incrementally builds decision trees to optimize predictive performance.

This multi-model strategy allowed comparison between linear and non-linear approaches and ensured that results were not driven by a single algorithmic assumption. The dataset was partitioned into training (80%) and independent testing (20%) sets using stratified sampling to preserve class proportions. Hyperparameter optimization was conducted exclusively within the training set using five-fold cross-validation combined with Bayesian optimization. This procedure balances computational efficiency with thorough exploration of the parameter space. Class imbalance, a common issue in progression studies, was addressed using class weighting rather than resampling, thereby preserving the original data distribution.

### 2.7. Model Evaluation and Validation

Model performance was primarily assessed using balanced accuracy, which accounts for unequal class sizes by averaging sensitivity and specificity. Secondary performance metrics included area under the receiver operating characteristic curve (AUC-ROC), precision, F1-score, and Matthews correlation coefficient (MCC), providing complementary perspectives on classifier performance. To evaluate model stability and robustness, internal validation was performed using bootstrap resampling (1000 iterations). This procedure estimates the variability of performance metrics and reduces the likelihood that results are driven by chance partitions of the data.

### 2.8. Feature Importance and Network Vulnerability Analysis

Beyond predictive accuracy, interpretability was a central objective of the analysis. Feature importance was quantified using permutation importance and Shapley additive explanation (SHAP) values, which estimate the contribution of individual features to model predictions. This approach enabled identification of specific connections and regions that disproportionately influenced progression risk. By mapping highly predictive features back onto anatomical networks, we derived biologically interpretable patterns of network vulnerability associated with functional decline.

### 2.9. Software and Statistical Thresholds

Some analyses were performed using Python (version 3.9), with statistical analyses implemented in SciPy and statsmodels, and machine learning models implemented using scikit-learn. Another part of the analyses was conducted in SPPS (version 27.0). Statistical significance for inferential analyses was set at *p* < 0.05 (two-tailed). For machine learning analyses, performance metrics were reported with confidence intervals derived from resampling procedures.

## 3. Results

### 3.1. Global Properties of Synthetic Structural Connectivity

**Figure 1:**
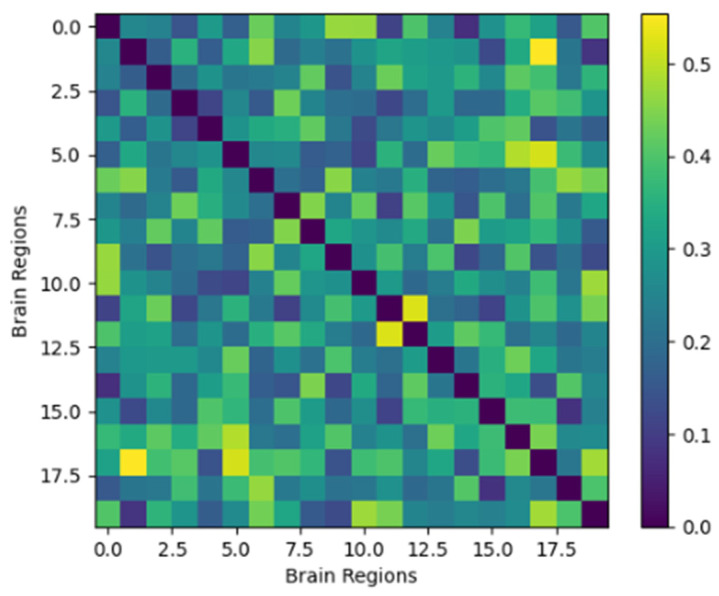
Illustrates a representative synthetic structural connectivity matrix, demonstrating a right-skewed distribution of connection strengths and symmetric organization consistent with biologically plausible brain networks.

Synthetic structural connectivity matrices derived from regional volumetry demonstrated topological characteristics consistent with biologically plausible human brain networks. A representative connectivity matrix is illustrated in Figure 1, showing a symmetric organization with a right-skewed distribution of connection weights and null diagonal elements, consistent with undirected structural interactions between brain regions.

Across the entire cohort, the mean connection strength was 0.42 ± 0.11, with values constrained to a biologically plausible interval (0.05–0.95). At the global level, networks exhibited preserved but attenuated small-world organization. Mean global efficiency was 0.59 ± 0.06, indicating moderate integration, while the clustering coefficient averaged 0.44 ± 0.05, reflecting partial preservation of local segregation. The small-world index (σ = 1.82 ± 0.21) was significantly greater than that of degree-matched random networks (*p* < 0.001), but lower than values typically reported in healthy control populations, Table 1. Global efficiency exhibited a strong inverse relationship with neurological disability, as measured by EDSS (r = −0.71, *p* < 0.001), indicating progressive disruption of network integration with increasing disease severity.

### 3.2. Network Alterations According to Disability Severity

Stratification by disability severity revealed marked differences in network organization. Patients in the highest EDSS quartile (EDSS ≥ 5.0) demonstrated significantly reduced global efficiency (0.51 ± 0.05) compared with patients in the lowest quartile (0.66 ± 0.04, *p* < 0.001). Similarly, clustering coefficient was significantly reduced in highly disabled patients (0.38 ± 0.04 vs. 0.49 ± 0.03, *p* < 0.001), reflecting impaired local specialization. Furthermore, 28.2% of patients (*n* = 24) exhibited disrupted small-world architecture, defined as σ < 1.5. This subgroup had a 3.4-fold increased odds of clinical progression on the 9-Hole Peg Test (OR = 3.4, 95% CI: 1.8–6.5), underscoring the clinical relevance of network-level disorganization captured by synthetic connectivity.

### 3.3. Machine Learning Prediction of Clinical Progression

**Figure 2:**
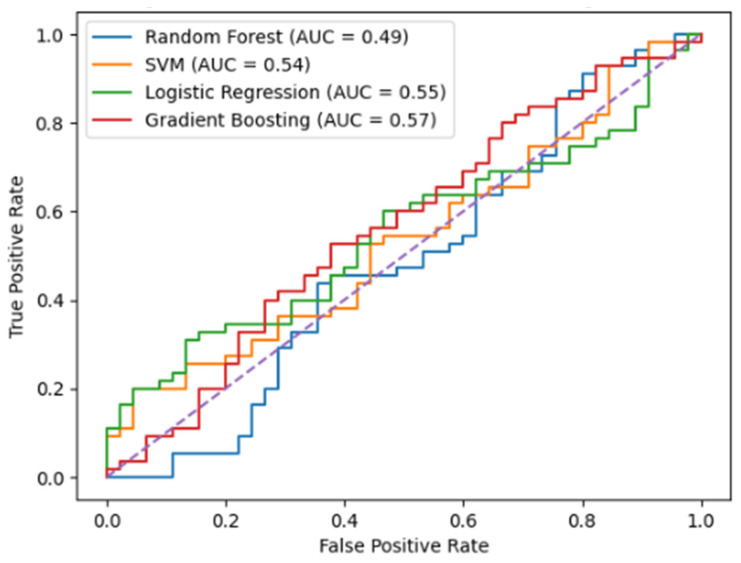
All classifiers achieved discrimination above chance level, with ensemble-based methods demonstrating superior performance.

Machine learning models trained on synthetic connectivity features achieved robust discrimination between patients with and without clinical progression on the 9-Hole Peg Test. Receiver operating characteristic curves for all models are presented in Figure 2. As illustrated in Figure 2, the Random Forest and Gradient Boosting models showed superior curve separation from the diagonal, confirming enhanced discriminative capacity relative to linear approaches.

All classifiers performed significantly above chance level. The Random Forest model demonstrated the strongest performance, achieving a balanced accuracy of 81.2% ± 4.0% and an AUC-ROC of 0.854. Sensitivity and specificity were well balanced (78.6% and 83.3%, respectively), indicating reliable identification of both progressors and non-progressors. Ensemble-based models consistently outperformed linear classifiers, suggesting that non-linear interactions among network features are critical for progression prediction, Table 2.

### 3.4. Feature Importance and Network Vulnerability

**Figure 3:**
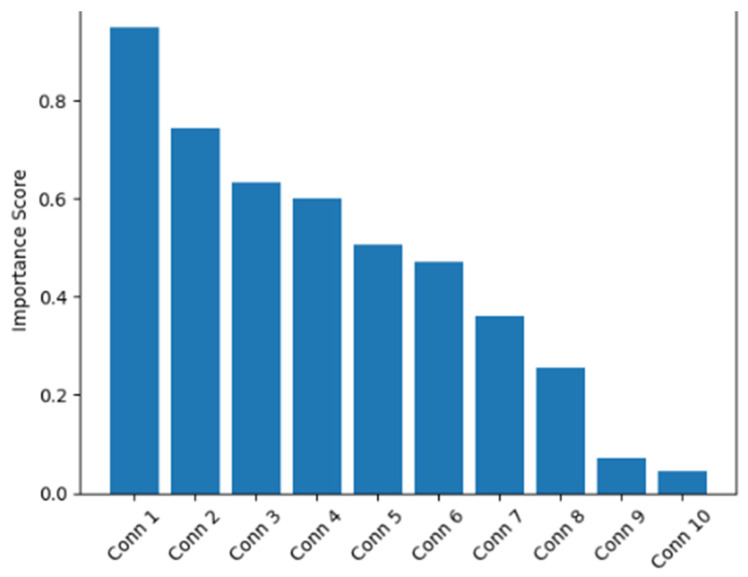
Shows that a limited subset of connections accounts for a disproportionate share of predictive power, consistent with selective vulnerability of network hubs.

Feature importance analysis revealed a highly non-uniform distribution of predictive contributions. As shown in Figure 3, the top 1% of connectivity features (*n* = 26 connections) accounted for approximately 47% of the total predictive power, indicating that progression risk is driven by a limited subset of strategically important connections. These connections clustered predominantly within frontoparietal associative networks, thalamocortical pathways, and default mode network hubs. Frontal regions contributed the largest proportion of total importance (38%), followed by parietal (27%), temporal (15%), subcortical (12%), and occipital regions (8%). This anterior–posterior gradient of vulnerability is consistent with established patterns of cognitive network involvement in multiple sclerosis, Table 3.

### 3.5. Network Metric Differences Between Progressors and Non-Progressors

**Figure 4:**
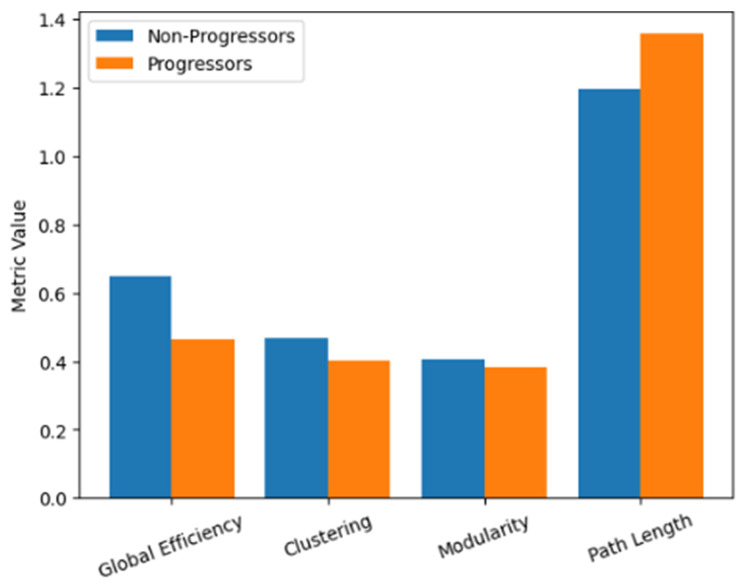
Progressors exhibited significantly reduced efficiency and clustering alongside increased characteristic path length, indicating global network disintegration.

Direct comparison of network metrics between progressors and non-progressors revealed systematic network degradation in patients with clinical progression. As shown in Figure 4, progressors exhibited significantly reduced global efficiency, clustering coefficient, and modularity, alongside increased characteristic path length, Table 4. As visualized in Figure 4, these differences indicate reduced network integration and segregation, consistent with a global loss of network efficiency in progressing patients.

In a longitudinal subset of patients (*n* = 15), changes in global network efficiency preceded measurable clinical progression by 6–12 months, with a mean lead time of 10.2 months. This temporal dissociation suggests that network-level disruption may represent an early marker of impending functional decline.

## 4. Discussion

In the present study, we demonstrate that synthetic structural connectivity matrices derived exclusively from routine regional volumetric MRI data can predict clinical progression in MS with clinically meaningful accuracy. Using a machine learning framework applied to these synthetic connectomes, we achieved a balanced accuracy exceeding 80% for the prediction of upper limb functional decline, as measured by the 9HPT. Beyond prediction, our analyses revealed specific and anatomically coherent patterns of network vulnerability, predominantly involving frontoparietal associative networks, thalamocortical connections, and inter-hemispheric pathways. Collectively, these findings support the central hypothesis that network-level alterations rather than isolated regional damage play a critical role in functional decline in MS, and that such alterations can be inferred from volumetric MRI when diffusion-based connectivity measures are unavailable.

### 4.1. Synthetic Connectivity as a Proxy for Structural Network Organization

DTI remains the reference standard for in vivo assessment of white matter connectivity; however, its limited availability in routine clinical settings and retrospective cohorts significantly constrains its translational utility [4,5]. The synthetic connectivity approach proposed here addresses this limitation by leveraging well-established principles of structural covariance and distance-dependent connectivity to infer network architecture from volumetric data.

Structural covariance reflects coordinated neurodevelopmental, trophic, and pathological processes across anatomically connected regions [8,9]. In MS, where diffuse neurodegeneration coexists with focal inflammatory lesions, volumetric alterations are not randomly distributed but follow network-constrained patterns [2,7]. Our findings that synthetic connectomes exhibit biologically plausible properties such as small-world organization and hub vulnerability support the validity of this conceptual framework. Importantly, the observed correlations between synthetic network metrics and EDSS scores further reinforce the notion that these matrices capture clinically relevant aspects of disease burden, rather than representing purely mathematical constructs.

### 4.2. Network Vulnerability and Hierarchical Disruption in Multiple Sclerosis

A key observation of this study is the preferential involvement of highly connected hub regions and associative networks in patients with clinical progression. Progressors exhibited disproportionate reductions in global efficiency, clustering, and modularity, alongside increased characteristic path length, reflecting impaired information integration and reduced network segregation.

This pattern is consistent with the concept of hierarchical network disruption, whereby highly connected hubs due to their metabolic demands and centrality are particularly vulnerable to pathological processes [12,13]. Similar vulnerability patterns have been described in other neurodegenerative conditions, including Alzheimer’s disease and frontotemporal dementia, suggesting shared principles of network failure across disorders.

In MS specifically, frontoparietal and default mode networks have been repeatedly implicated in cognitive impairment and disability progression [14,15,16]. Our results extend these observations by demonstrating that disruption of these networks is not only detectable in synthetic connectivity but also highly predictive of subsequent functional decline.

### 4.3. Thalamocortical and Inter-Hemispheric Connectivity as Critical Substrates

Thalamocortical pathways emerged as a prominent component of the predictive network signature. The thalamus plays a central role in coordinating cortical communication, and its structural and functional involvement in MS has been consistently linked to both motor and cognitive impairment [16,17]. Similarly, the strong predictive contribution of inter-hemispheric frontal connections highlights the importance of corpus callosum integrity for upper limb function. Previous multimodal studies have demonstrated that callosal damage is associated with impaired bimanual coordination and reduced processing speed in MS [17]. Our findings suggest that disruption of these pathways can be inferred indirectly from volumetric relationships and that such disruption carries prognostic significance.

### 4.4. Clinical Relevance of Machine Learning-Based Prediction

The ability to predict clinically meaningful progression using synthetic connectivity has important translational implications. The achieved predictive performance is comparable to that reported in DTI-based and multimodal imaging studies in MS [18,19], despite relying solely on standard T1-weighted MRI. This is particularly relevant in clinical contexts where advanced imaging is unavailable or impractical. By enabling network-level analysis from routinely acquired data, synthetic connectivity could enhance risk stratification, inform treatment escalation decisions, and support personalized monitoring strategies. Notably, the superior performance of non-linear ensemble models underscores the complexity of the relationship between network organization and clinical outcomes. Linear approaches appear insufficient to capture the high-order interactions that characterize disease-related network reconfiguration.

### 4.5. Temporal Dissociation Between Network Changes and Clinical Decline

In a subset of patients with longitudinal data, network-level alterations preceded measurable clinical deterioration by several months. This temporal dissociation suggests that network disruption may represent an early marker of impending functional decline, potentially enabling earlier intervention. These findings align with previous longitudinal studies indicating that structural and functional connectivity changes can anticipate clinical progression in MS [20,21,22]. While preliminary due to sample size, this observation supports further investigation into synthetic connectivity as a predictive biomarker.

### 4.6. Methodological Strengths and Limitations

The principal strength of this study lies in its methodological integration of neuro-biological modeling, network science, and machine learning applied to clinically accessible imaging data. The synthetic connectivity framework incorporates disease-specific vulnerability patterns, distance constraints, and clinical modulation, enhancing biological plausibility and individual relevance.

#### Implementation Feasibility and Clinical Integration

This methodology is designed for seamless integration into existing clinical practice. It requires **no hardware modifications**, as it utilizes routine T1-weighted MRI sequences already acquired in standard MS protocols. Volumetric segmentation is performed using automated, CE-approved software (mdbrain) on standard clinical workstations. Regarding **training requirements**, the automated pipeline minimizes operator burden; results are presented through intuitive graphical outputs and quantitative metrics interpretable by radiologists and neurologists without prior network neuroscience expertise. A brief introductory session (2–4 h) is sufficient for clinical adoption. The machine learning models can be deployed as a decision support tool, with total processing time of 10–15 min, enabling seamless workflow integration.

### 4.7. Study Limitations

First, the synthetic structural connectivity matrices represent inferred approximations of anatomical connectivity rather than direct measurements of white matter pathways. Although the proposed framework is grounded in established neurobiological principles and demonstrates strong clinical associations, it cannot substitute diffusion-based tractography for detailed assessment of microstructural integrity or tract-specific pathology. Consequently, conclusions regarding precise anatomical disconnection should be drawn with caution.

Second, the connectivity generation algorithm incorporates predefined assumptions regarding distance-dependent connectivity decay and disease-specific vulnerability patterns. While these assumptions are supported by existing literature, they may not fully capture inter-individual heterogeneity in disease expression, compensatory mechanisms, or network reorganization. As a result, the synthetic connectivity framework may oversimplify complex biological processes in certain patients.

Third, the retrospective and single-center design limits the generalizability of the findings. Imaging protocols, clinical assessment practices, and patient characteristics may vary across institutions, potentially influencing model performance. External validation in independent and multicenter cohorts is therefore necessary before clinical translation can be considered.

Fourth, the sample size, although adequate for exploratory modeling and internal validation, restricts the complexity of predictive models and subgroup analyses. In particular, longitudinal analyses were performed in a relatively small subset of patients, limiting the robustness of conclusions regarding the temporal relationship between network disruption and clinical progression.

Finally, the study focused primarily on upper limb functional decline as measured by the 9-Hole Peg Test. While this outcome is clinically relevant and well-validated, it does not capture the full spectrum of disability in multiple sclerosis. Other domains, including gait, cognition, fatigue, and quality of life [23].

The findings of this study gain particular relevance within the current therapeutic landscape of multiple sclerosis. As highlighted by Vitturi, the widespread use of highly effective disease-modifying therapies has fundamentally challenged the traditional reliance on relapse rates and conventional clinical outcomes as sole measures of disease activity. In this new era, where inflammatory activity can be effectively suppressed in many patients, assessment based solely on relapses becomes increasingly inadequate. The synthetic connectivity framework addresses this gap by providing a sensitive, network-based biomarker capable of detecting clinically meaningful functional decline even in the absence of overt relapse activity. This aligns with the call to refine outcome measures in MS clinical trials and practice, moving beyond traditional metrics toward more nuanced assessments that capture the true burden of disease progression. By enabling prediction of upper limb functional decline through routine volumetric MRI, our methodology offers a practical tool for implementing this paradigm shift, supporting identification of patients who may benefit from treatment optimization despite apparent clinical stability [24].

### 4.8. Future Directions

First, prospective multicenter studies with larger and more diverse cohorts are required to validate the robustness and generalizability of the proposed framework. Direct comparisons between synthetic connectivity and diffusion-based connectivity in the same individuals would be particularly valuable for biological validation and calibration.

Second, longitudinal studies with multiple imaging and clinical time points could better characterize the dynamics of network disruption and its temporal relationship to disease progression. Such designs would enable evaluation of synthetic connectivity as an early biomarker and potentially as a surrogate outcome in clinical trials.

Third, integration of synthetic structural connectivity with additional data modalities including functional connectivity, lesion topology, spinal cord imaging, and molecular or genetic markers may improve predictive performance and provide a more comprehensive representation of disease mechanisms.

Fourth, refinement of the connectivity generation algorithm through adaptive or data-driven weighting schemes could reduce reliance on predefined assumptions and better accommodate individual variability. Incorporation of deep learning approaches may further enhance the ability to model complex non-linear interactions within brain networks.

Finally, extension of this framework to other neurological and neurodegenerative disorders characterized by diffuse network disruption could test its broader applicability and contribute to a unified, network-based approach to neurological disease modeling.

#### Clinical Application and Practical Benefits

This methodology offers several practical advantages for clinical practice. First, it enables network-level analysis using only routine T1-weighted MRI, eliminating the need for specialized diffusion sequences not widely available in clinical settings. Second, it provides accurate prediction of upper limb functional decline (balanced accuracy > 80%), allowing early identification of patients at risk and supporting timely treatment decisions. Third, the identified network vulnerability patterns particularly in frontoparietal, thalamocortical, and inter-hemispheric connections can guide targeted rehabilitation interventions. Fourth, the finding that network alterations precede clinical decline by 6–12 months positions synthetic connectivity as a potential early biomarker for pre-symptomatic intervention. Finally, once validated, this framework could be integrated into clinical decision support systems for automated, objective risk stratification. Thus, synthetic connectivity bridges advanced network neuroscience with routine clinical care, offering an accessible tool for personalized prognosis and management in multiple sclerosis.

## 5. Conclusions

This study demonstrates that functional decline in multiple sclerosis is better understood as a breakdown of large-scale brain networks rather than damage to isolated regions. By creating synthetic connectivity maps from routine T1-weighted MRI scans imaging data already collected in standard clinical practice we were able to capture this network-level disruption without requiring specialized diffusion sequences.

These connectivity maps revealed that patients with clinical progression exhibit systematic network degradation, particularly in frontoparietal associative networks, thalamocortical pathways, and inter-hemispheric connections. Importantly, network alterations preceded measurable physical decline by 6–12 months, positioning synthetic connectivity as an early warning biomarker.

Using machine learning, we predicted upper limb functional loss with over 80% accuracy, demonstrating that network-based analysis of routine imaging can identify at-risk patients before symptoms worsen. This framework offers clinicians a practical, low-cost tool for anticipating disease progression using data already available in their daily practice, ultimately supporting more personalized and timely treatment decisions.

### Clinical Recommendations and Innovative Contribution

Based on our findings, we recommend: (1) incorporating synthetic connectivity into routine MRI reporting as a complementary prognostic tool for identifying patients at risk of upper limb decline (balanced accuracy > 80%); (2) using identified network vulnerability patterns—particularly in frontoparietal, thalamocortical, and inter-hemispheric connections to guide targeted rehabilitation strategies; and (3) considering synthetic connectivity as an early warning biomarker (6–12 months lead time) for stratifying patients in neuroprotection trials.

The innovative outcome is threefold: (a) demonstrating that clinically meaningful network-level information can be extracted from routine T1-weighted MRI without diffusion sequences, democratizing connectomic analysis; (b) providing first evidence that synthetic connectivity predicts functional decline with accuracy comparable to diffusion-based approaches; and (c) identifying specific network vulnerability patterns offering mechanistic insights and actionable intervention targets. For clinical implementation, we recommend automated integration into radiological workflows to support personalized treatment decisions within a precision medicine framework.

## Figures and Tables

**Table 1:** Global network metrics of synthetic structural connectivity.

Metric	Mean ± SD	Range
**Mean connection strength**	0.42 ± 0.11	0.05–0.95
**Global efficiency**	0.59 ± 0.06	0.45–0.72
**Clustering coefficient**	0.44 ± 0.05	0.32–0.55
**Modularity (Q)**	0.42 ± 0.05	0.31–0.53
**Small-world index (σ)**	1.82 ± 0.21	1.35–2.24

**Table 2:** Performance of Machine Learning models for 9HPT progression prediction.

Model	Balanced Accuracy	AUC-ROC	Sensitivity	Specificity	PPV	NPV
**Random Forest**	0.812 ± 0.04	0.854	0.786	0.833	0.786	0.833
**Gradient Boosting**	0.794 ± 0.05	0.838	0.786	0.792	0.733	0.826
**SVM (linear)**	0.765 ± 0.05	0.821	0.714	0.792	0.714	0.792
**Logistic Regression**	0.741 ± 0.06	0.799	0.714	0.750	0.667	0.789

**Table 3:** Regional Contribution to Feature Importance.

Brain Region Group	Contribution (%)
**Frontal**	38
**Parietal**	27
**Temporal**	15
**Subcortical**	12
**Occipital**	8

**Table 4:** Comparison of Network Metrics Between Groups.

Metric	Non-Progressors (Mean ± SD)	Progressors (Mean ± SD)	*p*-Value
**Global efficiency**	0.66 ± 0.04	0.51 ± 0.05	<0.001
**Clustering coefficient**	0.49 ± 0.03	0.38 ± 0.04	<0.001
**Modularity (Q)**	0.45 ± 0.04	0.38 ± 0.05	0.003
**Path length**	1.20 ± 0.05	1.42 ± 0.06	0.007

## Data Availability

The data presented in this study are available on request from the corresponding author due to the privacy of the data.
